# Impact of Er:YAG laser on wound healing following nonsurgical therapy: A pilot study

**DOI:** 10.1002/cre2.179

**Published:** 2019-04-02

**Authors:** Kandice L. Klepper, Yong‐Hee Patricia Chun, David Cochran, Shuo Chen, Howard S. McGuff, Brian L. Mealey

**Affiliations:** ^1^ Department of Periodontics UT Health San Antonio San Antonio Texas USA; ^2^ Department of Cell Systems and Anatomy UT Health San Antonio San Antonio Texas USA; ^3^ Department of Developmental Dentistry UT Health San Antonio San Antonio Texas USA; ^4^ Department of Biomedical Sciences UT Health San Antonio San Antonio Texas USA

**Keywords:** laser, nonsurgical periodontal therapy, periodontal disease, wound healing

## Abstract

The purpose is to examine early wound healing through histological analysis by characterizing connective tissue distribution and organization in the treated periodontium following nonsurgical therapy. Periodontal disease is a multifactorial pathological process that leads to the loss of the surrounding periodontium. Traditional periodontal therapies have proven beneficial in halting the progression of disease. The aim of this study is to investigate early wound healing in periodontal patients following hand/ultrasonic instrumentation alone, erbium‐doped yttrium aluminum garnet laser instrumentation alone, or a combination of hand/ultrasonic instrumentation and Er:YAG laser instrumentation for the nonsurgical treatment of periodontitis by histologic evaluation. Twenty‐one patients were randomized to receive nonsurgical therapy for the treatment of chronic periodontitis with three modalities prior to surgical therapy. Baseline clinical measurements were obtained prior to treatment. Wound healing was assessed by obtaining an otherwise discarded tissue sample following nonsurgical therapy of the selected study site. Samples were obtained at 2 or 6 weeks following initial therapy with a step‐back incision and fixated for histological and immunohistochemical analysis. There were minimal between‐group differences in the amount of collagen distribution when analyzing the Mallory–Heidenhain Azan trichrome, Picrosirus Red stain, and proliferating cell nuclear antigen at both time points. Descriptive analysis of baseline measurements showed no differences in probing depth change, bleeding on probing, and clinical attachment level following initial therapy between the three treatment groups at 2 or 6 weeks. Each treatment modality was effective in treating moderate to severe chronic periodontitis; however, the results of this study are inconclusive regarding superiority of any one treatment approach from a histologic and immunohistochemical perspective. Based on this assessment, there was increased fibroblast proliferation and collagen maturation between the 2‐ and 6‐week time point after treatment in all treatment groups, with few apparent differences between treatment groups. This pilot study qualitatively evaluated early wound healing in periodontal patients following non surgical therapy with various treatment modalities. When comparing descriptive outcomes of Er:YAG laser therapy and hand/ultrasonic instrumentation there were minimal differences in collagen distribution and density between groups. The evaluated modalities were each effective treating periodontal patients with non surgical therapy.

## 1 INTRODUCTION

The prevalence of periodontitis in the United States is 47%, and even more common with increasing age (Eke et al., 2015). Periodontal disease has many etiological factors that contribute to the breakdown of the oral environment, including a shift in the subgingival microflora with a corresponding immunoinflammatory response (Genco, 1993). Traditional periodontal therapies have proven beneficial in halting the progression of disease. These methods include debridement of the root surface by scaling and root planing (SRP) to disrupt the biofilm and remove calculus.

The most commonly used methods of nonsurgical therapy involve use of hand scalers/curettes and ultrasonic instruments to debride the root surface; this is known as SRP (Eberhard, Jervoe‐Storm, Needleman, Worthington, & Jepsen, 2008; Hung & Douglass, 2002). Previous literature suggests clinicians can expect approximately 2‐mm reduction in probing depths and 1‐mm gain in clinical attachment levels when initial probing depths were greater than 6 mm^4^. More recent clinical studies have examined the use of laser therapy, including the Er:YAG laser, as an alternative or adjunctive form of SRP, as some types of laser therapy may have inherent hemostatic, antibacterial, and ablation properties (Cobb, 2006).

The Er:YAG (erbium‐doped:yttrium‐aluminum‐garnet) laser utilizes infrared pulsations at 2,940 nm to safely remove plaque and calculus while reducing bacterial biofilm from the root surface (Frenzten, Braun, & Aniol, 2002). Overall, Er:YAG treatment in periodontal therapy includes disinfection and detoxification through its bactericidal nature, ability to inactivate endotoxin, and proposed biostimulatory effects to promote wound healing promotion; in addition, the Er:YAG laser has minimal thermal effects (Aoki et al., 2015). The results of a systematic review comparing the use of SRP therapy, combined SRP + Er:YAG, and Er:YAG alone found no significant difference in clinical attachment gain or probing depth reduction at 6 and 12 months (Sglostra, Pertucci, Gatto, & Monaco, 2012). All modalities resulted in a significant reduction in overall probing depth and bleeding on probing, which suggest laser treatment as an appropriate alternative to hand instrumentation and ultrasonic instruments for nonsurgical therapy in periodontal disease (Schwartz, Aoki, Becker, & Sculean, 2008).

An examination of the histological and immunohistochemical wound healing events in humans, which take place following use of both techniques, has yet to be clearly determined. The characterization of wound healing following nonsurgical therapy was evaluated in dogs and proposed that Er:YAG application may influence the establishment of new connective tissue attachment to the root surface following treatment of periodontal disease (Schwartz et al., 2007). The aim of the current study was to further investigate early wound healing in periodontitis patients following nonsurgical therapy using hand/ultrasonic instrumentation alone, Er:YAG instrumentation alone, or a combination of hand/ultrasonic instrumentation and Er:YAG instrumentation for the nonsurgical treatment of chronic periodontitis. The objective was to histologically evaluate early wound healing by characterizing connective tissue organization, and cellular proliferation in the treated periodontium.

## 2 MATERIALS AND METHODS

### 2.1 Participant enrollment

The University of Texas Health Sciences Center at San Antonio Institutional Review Board (UTHSCSA IRB: # HSC20150672H) approved this pilot study. A power analysis was not conducted as the goal of this study was to analyze the healing response qualitatively. Enrolled patients needed to meet the following inclusion criteria: age ≥ 18, diagnosed with moderate to severe chronic periodontitis requiring both nonsurgical and surgical therapy, and one or more sites with ≥6 mm of probing depth and bleeding on probing. All patients enrolled signed informed consent prior to beginning the study. Exclusion criteria consisted of the following: pregnant women or women planning to become pregnant, current smokers, any systemic condition or active infection that would impair wound healing (i.e., diabetes and autoimmune disease), history or current use of chemotherapeutic or immunosuppression medication, and previous periodontal therapy.

### 2.2 Nonsurgical and surgical protocol

Patients were grouped by convenience sample to receive nonsurgical therapy with hand/ultrasonic instrumentation alone (Group A), Er:YAG laser alone (Group B), or a combination of hand/ultrasonic instrumentation and Er:YAG laser therapy (Group C). Calibrated investigators obtained clinical measurements of probing depth, distance of gingival margin to cemento‐enamel junction, and bleeding status were made at baseline and again at either 2 or 6 weeks following initial therapy. In the 2‐week time point, Group A enrolled three patients, Group B enrolled five patients, and Group C enrolled two patients. In the 6‐week time point, Group A enrolled two patients, Group B enrolled three patients, and Group C enrolled three patients. The selected study sites included only posterior teeth with PD ≥6 mm, which would require further periodontal surgery following nonsurgical therapy. Selected sites were treated nonsurgically with either of the three treatment modalities. The AdvErL Er:YAG laser (Morita USA; Irvine, CA) was standardized with settings of emission at 2,940 nm with 25 Hz (pulses per second), 80 mJ and water spray. For each patient, the amount of time required to treat the root surface was recorded. All patients received oral hygiene instruction to ensure adequate plaque control prior to surgical therapy. None of the patients received antibiotic treatment prior to either nonsurgical or surgical periodontal therapy.

Surgical therapy was performed only at sites with residual probing depth of ≥4 mm and bleeding on probing (all probing depths prior to surgery were at least 5 mm). Following administration of local anesthesia, a step‐back incision was made, and 1‐ to 3‐mm collar of diseased marginal tissue was excised and stained with India ink on the oral epithelial surface. The tissue samples were transferred to chromic gut suture paper with the junctional/sulcular epithelium facing the paper for orientation. Samples were then placed into an embedding cassette between two sponges and placed in Bouin's buffer Electron (Microscopy Sciences, Hartfield, PA) for fixation. After removal of the tissue sample, full thickness flaps were reflected, and periodontal defects were treated as indicated.

### 2.3 Histologic and immunohistochemical processing

After 24 hr of fixation with Bouin's buffer, tissue samples were washed with phosphate‐buffered saline prior to embedding. Samples were embedded in paraffin wax with the Leica EG 1160 Paraffin Embedding Center (Leica Biosystems, Buffalo Grove, IL). Sections were obtained using the Leica RM 2155 automatic microtome (Leica Biosystems, Buffalo Grove, IL). All samples were sectioned at 5‐μm thickness and placed on glass slides for staining.

The following stains were used to evaluate wound healing, according to the manufacturer's guidelines for qualitative connective tissue characterization: Mallory–Heidenhain Azan (Electron Microscopy Sciences, Hartfield, PA) and Picrosirius Red Stain + imaged under plane polarized light (Polysciences, Inc., Warrington, PA; Foster, 2012; Sawabe et al., 2015). Proliferating cell nuclear antigen (PCNA) was utilized for immunhistochemical analysis to evaluate fibroblast proliferation in the connective tissue (Häkkinen, Uitto, & Larjava, 2000). Wound healing was assessed qualitatively by the distribution, orientation, and density of connective tissue and degree of fibroblast proliferation. The slices were evaluated using light microscopy at 4× magnification, and digital images were obtained using CellSens software (Version 1.4 Software, Olympus America, Center Valley, PA). In addition, Picrosirius Red stained tissues were examined under plane polarized light by rotating slides to capture collagen birefringence. The samples included in the analysis are representative of each treatment group at both time points. The obtained sample images are oriented with the epithelial surface on the left and the previously attached tooth surface on the right for standardization.

### 2.4 Statistical analysis

Clinical measurements obtained at baseline and prior to surgical therapy were compared between groups and time points using descriptive statistics of means and standard deviations. Instrumentation time between groups was also compared during descriptive statistics. Due to the small number of patients in each group and time point in this pilot study, formal statistical analysis was not performed.

## 3 RESULTS

**Figure 1:**
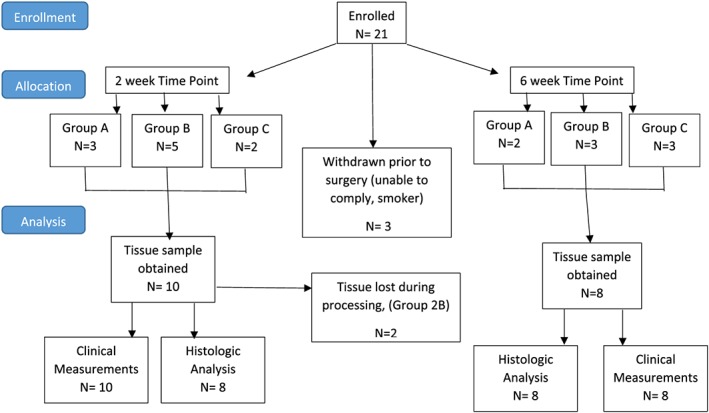
Study flow diagram. This flow diagram depicts events following patient recruitment through the completion of the study and illustrates details pertaining to exited patients and lost tissue samples during the study period

Sixteen of 21 enrolled patients completed the pilot study (Figure 1). The 21 enrolled patients included 13 males and 8 females, with a mean age of 60 and a range of 43 to 77 years; currently, there is no literature to suggest a difference in wound healing outcomes between patients in this age range. Both time points had a total of eight completed subjects. The enrolled patients were systemically healthy with no history of diabetes or other condition that would impair wound healing. All selected study sites were diagnosed with moderate to severe chronic periodontitis and received nonsurgical therapy. Of these 16 completed patients, representative samples were selected from each group and utilized for further descriptive analysis of early wound healing. Throughout the course of the study, neither adverse events nor signs of postoperative infection occurred. Following both nonsurgical and surgical treatment, all patients experienced normal clinical healing.

### 3.1 Mallory–Heidenhain Azan

**Figure 2:**
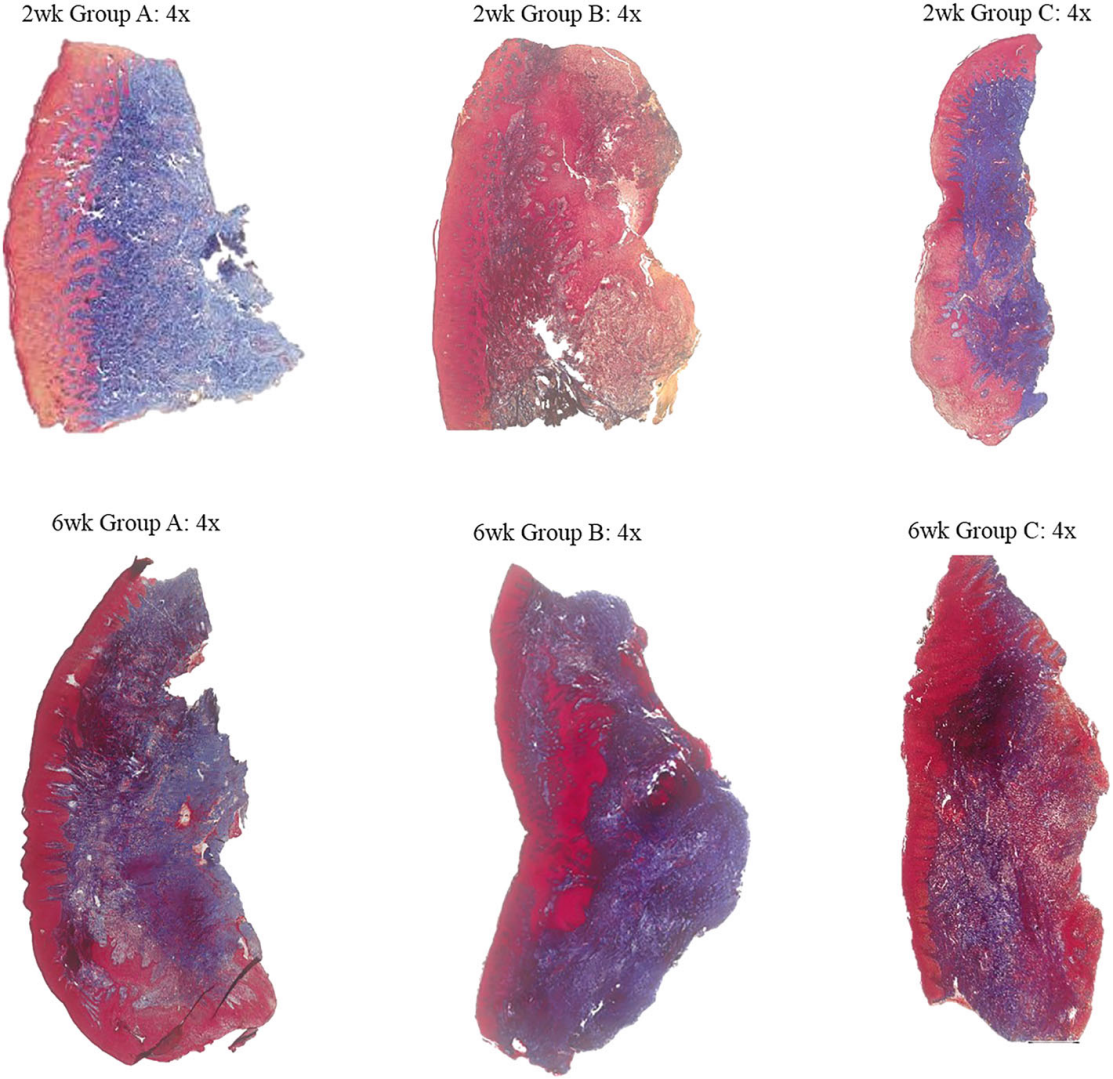
Comparison: 2‐ versus 6‐week time points: Mallory–Heidenhain Azan. 4× magnification—Group A: hand/ultrasonic instrumentation alone (scrp), Group B: Er:YAG laser treatment alone, and Group C: combination therapy (hand/ultrasonic instrumentation + Er:YAG laser)

This trichrome stain is used to characterize morphology and to distinguish various cells and tissues. This stain is primarily used to stain collagen, which appears blue; in contrast, nuclei are stained red (Sawabe et al., 2015). At the 2‐week time point, there were minimal differences in the amount of connective tissue between treatment groups. However, the distribution of collagen in each sample appeared very similar on higher magnification, taking into consideration the differences in size of the tissue samples. In qualitatively assessing the 6‐week time point, by comparing treatment groups, the amount of connective tissue was evenly distributed through each sample, which is evident by the distribution of red and blue staining throughout. When comparing the two time points, there was no difference in connective tissue distribution and orientation (Figure 2).

### 3.2 Picrosirius Red staining with plane polarized light filtration

**Figure 3:**
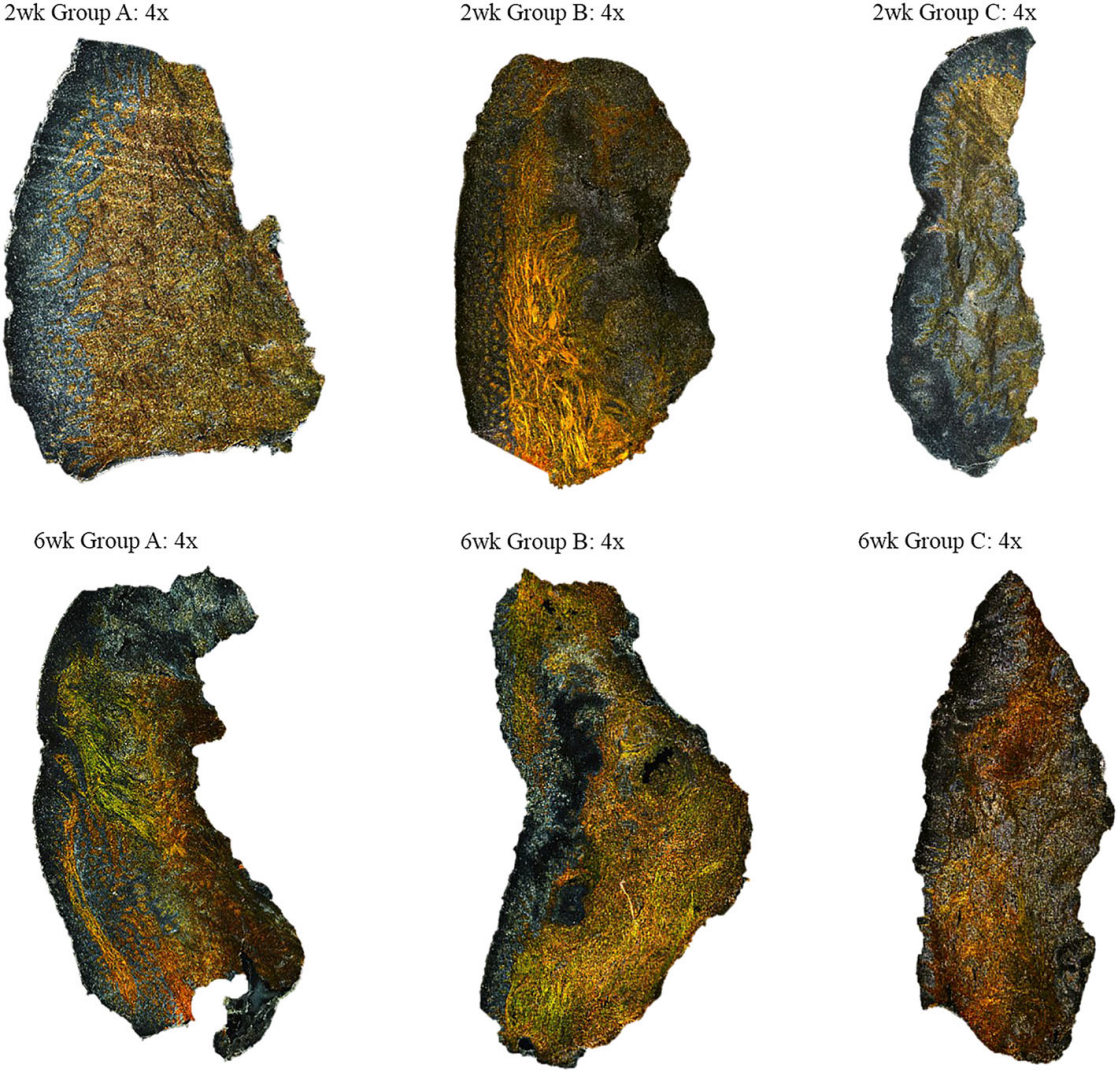
Comparison: 2‐ versus 6‐week time points: Picrosirius Red with plane polarized light filter. 4× magnification—Group A: hand/ultrasonic instrumentation alone (scrp), Group B: Er:YAG laser treatment alone, and Group C: combination therapy (hand/ultrasonic instrumentation + Er:YAG laser)

This stain requires rotation of two polarizing filters, which only allow polarized light to pass through the sample. Polarized light enhances the samples' birefringence, allowing for differentiation of the density and quantity of fibers, which is noted by color differentiation. Thick and densely packed collagen Type I is represented by vibrant yellow to red hues, whereas collagen Type III and loosely consolidated fibrils appear green (Sawabe et al., 2015). Regarding the 2‐week time point samples, there were minimal observed differences between treatment groups. Overall, the birefringence ranged from yellow to red indicating loosely arranged collagen also organized in various direction of fiber orientation. No other notable differences in collagen density and organization were observed between treatment groups in the 2‐week samples (Figure 3). In comparison to 2‐week samples, the birefringence in the 6‐week time point showed increased density of loosely organized collagen with well‐formed fiber bundles of yellow and orange coloration in addition to some interspersed thinner green fiber bundles. The arrangement of the collagen appeared elongated with some variation in fiber orientation. With regard to the selected representative samples, there were slight color differences in the 6‐week time point; however, these between‐group differences were negligible.

### 3.3 Proliferating cell nuclear antigen (PCNA)

**Figure 4:**
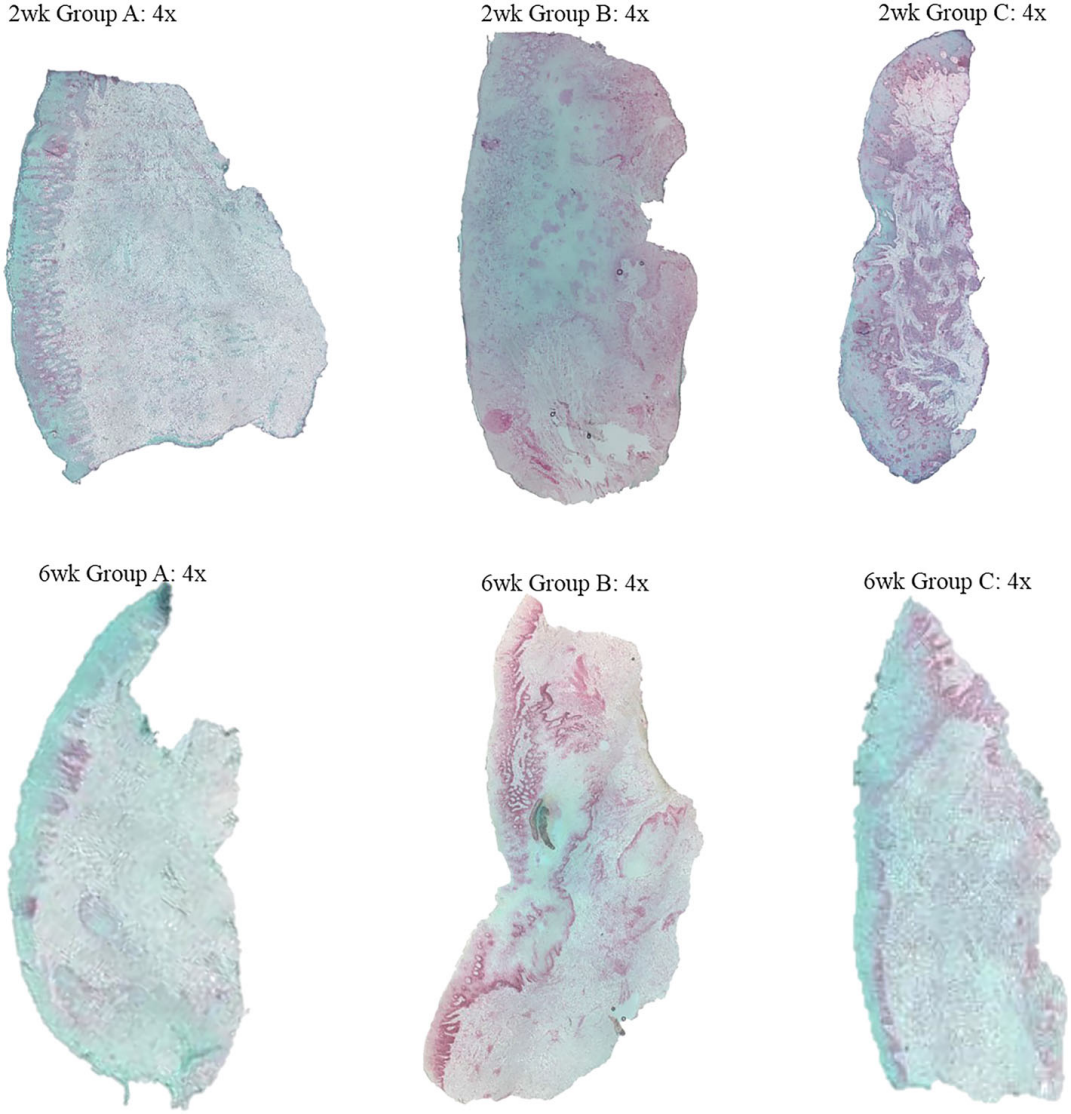
Comparison: 2‐ versus 6‐week time points: proliferating cell nuclear antigen. 4× magnification—Group A: hand/ultrasonic instrumentation alone (scrp), Group B: Er:YAG laser treatment alone, Group C: combination therapy (hand/ultrasonic instrumentation + Er:YAG laser)

This immunohistochemical technique is used to evaluate cellular activity, specifically in the G1 phase of the cell cycle. The red color observed, following sample preparation, indicates increased immunolocalization of the anti‐PCNA signal representing cellular proliferation. In the 2‐week time point (Figure 4), there was concentrated immunolocalization of nuclei undergoing cell division along the basal layer of the rete pegs; this is consistent with epithelial proliferation. On higher magnification, immunolocalization was also present and interspersed within the connective tissue indicating proliferation of fibroblasts. Overall, there were minimal differences in the included selective representative samples between treatment groups at the 2‐week time point. In comparison, the 6‐week time point exhibited expression of nuclear proliferation in the basal layer of the epithelium as seen in the innermost portion of the rete pegs. As with samples from the 2‐week time point, there were also minimal differences between treatment groups in the samples at 6 weeks (Figure 4).

Operating time ranged from 5 to 11 min, there was no major difference in treatment times between groups, with the combination therapy group having the longest treatment time (Tables 1 and 2). All study sites healed within normal limits following nonsurgical and surgical therapy; there were no appreciable differences in probing depth reduction, or gain in clinical attachment level (CAL) between groups and time points (Tables 1 and 2). The calculated mean probing depth reduction at 2 weeks following initial therapy ranged from 0.50 to 0.60 mm (Table 1). In contrast, the 6‐week time point exhibited slightly higher mean probing depth reduction of 1 .0mm as compared with the 2‐week time point (Table 2). There were notable differences between groups in the CAL gain at both the 2‐ and 6‐week time points (Tables 1 and 2). At 2 weeks, the mean CAL gain ranged from 0.17 to 1 .0mm (Table 1) whereas the mean CAL gain at 6 weeks ranged from 0 to 1.33 mm (Table 2). The number of subjects in this pilot study was insufficient to do meaningful statistical analyses on the secondary clinical outcomes.

**Table 1:** Clinical observations: 2‐week time point

Group	*N*	Mean treatment time (min) ± STD	SCRP (min)	Laser (min)	Mean change in probing depth (mm) ± STD	Mean change in clinical attachment level (mm) ± STD
2A	3	8.67 ± 5.69	8.67	NA	0.50 ± 0.50	0.17 ± 1.04
2B	5	10.00 ± 4.12	NA	10.00	0.60 ± 0.50	0.60 ± 0.50
2C	2	11.00 ± 0.82	5.25	5.75	0.50 ± 0.71	1.00 ± 1.41

STD: standard deviation; SCRP: scaling and root planing (non surgical therapy).

**Table 2:** Clinical observations: 6‐week time point

Group	*N*	Mean treatment time (min) ± STD	SCRP (min)	Laser (min)	Mean change in probing depth (mm) ± STD	Mean change in clinical attachment level (mm) ± STD
6A	2	8.00 ± 1.41	8.00	NA	1.00 ± 1.41	0.00 ± 0.00
6B	3	6.33 ± 1.53	NA	6.33	1.00 ± 1.73	1.33 ± 2.08
6C	3	10.33 ± 2.52	4.67	5.67	1.00 ± 1.00	0.67 ± 1.15

STD: standard deviation; SCRP: scaling and root planing (non surgical therapy).

## 4 DISCUSSION

The aim of this study was to qualitatively evaluate histologic early wound healing following nonsurgical therapy with various treatment modalities, specifically regarding the organization of collagen and cellular proliferation within the periodontium. Many methods have been used to treat the diseased root surface, and there is little difference in the histologic and treatment outcomes between the modalities used in this study (Smiley et al., 2015). Er:YAG laser, in comparison to traditional hand/ultrasonic instrumentation, has been shown to improve periodontal healing following nonsurgical therapy (Schwartz et al., 2008; Smiley et al., 2015).

In this pilot study, the primary objective of histologic and immunohistochemical gingival tissue healing was assessed at two time points following nonsurgical therapy with differing treatment modalities. Histologic early wound healing was evaluated by characterizing collagen distribution, organization, and maturation in addition to the presence of proliferating cellular nuclear antigen in fibroblasts. As previously noted, there were no marked differences in the collagen density and fiber orientation in comparing the 2‐ and 6‐week groups with the Mallory–Heidenhain Azan stain. In reviewing the 2‐week samples, there are negligible differences in the coloration of the stain between treatment groups. The first obtained study samples were not stained in the same batch as the other included representative samples and could explain the variation in coloration. In addition, there was variability in tissue orientation as evidence by predominate rete pegs within the connective tissue and epithelium surrounding the sample. However, on higher magnification, the collagen distribution and density appear to be similar in each of the treatment groups regardless of orientation or coloration. The fibers appear to be elongated and loosely arranged at the 2‐week time point. In comparison, the 6‐week time point displayed densely packed collagen with irregular orientation indicating increased maturation. This finding is consistent with the literature suggesting increased maturation of collagen with increased healing time (Caton, Proye, & Polson, 1982).

There was a slight increase in birefringence in the Er:YAG laser‐alone group compared with the other treatments, as demonstrated by increased red coloration that indicates increased collagen density. In addition, the collagen fibers in the Er:YAG alone group appeared to be more consolidated with irregular orientation indicating increased density in organization. In the 6‐week hand/ultrasonic instrumentation group, the yellow‐orange coloration of the collagen appeared more loosely organized. The collagen in the Er:YAG laser alone and combination therapy appeared to have slightly increased yellow‐red coloration; however, the differences between groups were minimal. The variation amongst coloration and intensity of birefringence is a result of varying organization of collagen fiber types and clustering of fibrils. In addition, these findings indicate that, with time, collagen density and maturation increased. Due to the small number of samples, it is difficult to extrapolate these results to form definitive conclusions regarding collagen maturation and fiber organization.

In comparing both time points and treatment groups, there were minimal differences in the localization of anti‐PCNA, representing cellular proliferation. There was evidence of fibroblast proliferation within the connective tissue in all samples. Overall, samples in the 2‐week time point revealed even distribution of cellular proliferation in the connective tissue with minimal clustering, whereas the 6 week samples in all groups exhibited increased immunolocalization throughout, with some displaying localized concentrations of fibroblasts on the previous attached tooth surface.

It has been proposed, through the provided wavelength, that laser irradiation increases the reparative process of wound healing (Sawabe et al., 2015). Such outcomes are attributed to its direct bactericidal effects and ablation of diseased tissues. In addition, the Er:YAG laser can decontaminate root surfaces through microexplosions within the periodontal pocket and has been known to cause biostimulation of soft tissue (Ogita et al., 2015). In the current study, the results are not conclusive that the Er:YAG laser increases collagen maturation and fibroblast proliferation following nonsurgical therapy.

The secondary outcome in this study was to evaluate the clinical periodontal parameters following nonsurgical therapy. Overall, there was a greater reduction in probing depth at the 6‐week time point when compared with 2 weeks. This could be explained by the increased time required for complete resolution of inflammation following nonsurgical therapy. During probing, the probe tip may penetrate further into the connective tissue at the base of the periodontal sulcus at sites with inflammation of immature healing tissue, resulting in deeper probing depths than may be found with more advanced healing and decreased inflammation (Fowler, Garrett, Crigger, & Egelberg, 1982). Following removal of local factors and resolution of inflammation, there is re‐establishment of the epithelial attachment and connective tissue apparatus (Taylor & Campbell, 1972), after which the probe does not penetrate into the connective tissue apparatus and there is an overall reduction in the probing depth (Fowler et al., 1982).

The time points in this study were selected to evaluate the early stages of wound healing following differing treatment modalities. Evidence suggests that 1 week following Er:YAG laser ablation, loosely organized collagen is evident with complete re‐epithelization (Kesler, Koren, Kesler, Kristt, & Gal, 2000). Previous literature suggests most soft tissue wound healing is complete by 4 weeks (Caton et al., 1982). Though some minor differences were noted in analysis of the 2‐ and 6‐week samples in the current study, the differences were not distinct. This is consistent with the literature suggesting most early wound healing occurs within the first 2 weeks following treatment. In order to better assess early wound healing, future studies could include earlier time points after treatment such as 3 days and 1 week. These time points would be useful to evaluate and further qualitatively assess the resolution of inflammation and peak fibroblast proliferation, which commonly occur within the first 2 weeks of gingival healing (Häkkinen et al., 2000; Taylor & Campbell, 1972).

Throughout the duration of this pilot study, the materials and methods were constantly evolving with each patient enrolled. Because the current study is the first human histologic and immunohistochemical analysis using various nonsurgical treatment methods including Er:YAG laser therapy, much of the methodology changed in order to obtain quality samples. The first obtained marginal tissue samples were placed directly into Bouin's buffer for fixation; upon analysis, it was noted that there was alteration in the tissue such as rolling of the sample, and difficulty in orientation. The outlined sampling process, as listed in Section 2, was finalized as the study progressed to include use of an embedding cassette for obtained tissues. In addition, the initial tissue samples were cut to a thickness of 3 μm; after microscopic evaluation, it was noted the samples were tearing and the applied stains were not as vibrant as expected. The thickness of obtained tissue slices was increased from 3 to 5 μm to correct this outcome. Many of the smaller size samples collected early in the study were inadequate in size to cut new slices at the 5‐μm thickness for restaining and reanalyzing. This decreased the number of quality samples for final analysis.

With the evolution of the methodology, multiple rounds of histologic and immunohistochemical staining were performed in order obtain vibrant coloration within the samples for quality analysis. The final standardization of tissue preparation consisted of the materials and methods previously described: fixation for 12 to 24 hr in Bouin's buffer after placement of tissue samples on chromic gut paper in a sponge‐lined embedding cassette, followed by 5‐μm sections of the embedded tissue. Following standardization, the highest quality tissue samples were analyzed, and selective samples included in the results were representative of each of the treatment groups and time points. These final samples were then restained in order to ensure a more consistent coloration.

Limitations of this study include lack of a fixed reference point when obtaining the tissue samples. This study was approved to only obtain an otherwise discarded tissue sample. Due to differences in the type of surgical procedures being performed in different subjects and the incision technique required, tissue samples could not be standardized. Samples were harvested from different sites within the periodontium. The size of the tissue samples varied depending on the amount of step back in the incision design. Some samples were taken from the papilla, some from the line angle, and others from either the buccal or palatal aspect of the tooth. Thus, it was challenging to standardized tissue preparation and orientation for embedding. Therefore, as expected, collagen fiber orientation was not consistent amongst all samples. In future study designs, it would be beneficial to include the extraction of periodontally hopeless teeth along with a soft tissue collar to ensure proper histologic orientation. A larger sample size with multiple tissue samples for histologic and immunohistochemical analysis would also be beneficial for a comparative analysis. In the current study, the best representative sample from each treatment group at each time point was chosen for analysis. Despite the lack of significant differences between samples, because the number of samples analyzed was small, it is not possible to determine definitively whether or not use of Er:YAG laser therapy alone or in combination with hand/ultrasonic instrumentation has an impact on early wound healing following nonsurgical therapy.

Based on the findings of this pilot study, each method of nonsurgical therapy was effective in promoting the resolution of inflammation in order to restore health in the periodontium. At 2 weeks, there were varying stages of collagen maturation and fiber organization within the tissues; this was evident in the histologic staining. The PCNA analysis revealed epithelial and fibroblast proliferation as indicated by immunolocalization in the samples. These findings were also apparent at 6 weeks, with increased collagen maturation, fiber organization, and cellular immunolocalization. In conclusion, compared with hand/ultrasonic instrumentation alone, there were negligible differences in Er:YAG laser alone or in combination with hand/ultrasonic instrumentation regarding increased collagen density, maturation, and fibroblast proliferation at 2 and 6 weeks following initial therapy. Lastly, there were no marked clinical differences in either time point between treatment groups.

## CONFLICT OF INTEREST

None declared.

## FUNDING INFORMATION

No funding information provided.
